# Pediatric Mental Health Emergency Visits During the COVID-19 Pandemic

**DOI:** 10.2478/sjcapp-2022-0005

**Published:** 2022-06-12

**Authors:** Daniel Hernández-Calle, Jorge Andreo-Jover, Javier Curto-Ramos, Daniel García Martínez, Luis Vicente Valor, Guillermo Juárez, Margarita Alcamí, Arancha Ortiz, Noelia Iglesias, María Fe Bravo-Ortiz, Beatriz Rodríguez Vega, Gonzalo Martínez-Alés

**Affiliations:** 1 Department of Psychiatry, Clinical Psychology and Mental Health, La Paz University Madrid, Spain; 2 Hospital La Paz Institute for Health Research (IdiPAZ), Madrid, Spain; 3 Department of Psychiatry, Universidad Autónoma de Madrid (UAM), Madrid, Spain; 4 Centro de Investigación Biomédica en Red de Salud Mental (CIBERSAM), Madrid, Spain; 5 Department of Epidemiology, Columbia University Mailman School of Public Health, New York, NY, US; 6 CAUSALab, Harvard University T.H. Chan School of Public Health, Boston, MA, US

**Keywords:** COVID-19, psychiatric urgency, paediatric mental health, suicide

## Abstract

**Introduction:**

Paediatric and adult psychiatric emergency department (ED) visits decreased during the initial COVID-19 outbreak. Long-term consequences of the COVID-19 pandemic will include increases in mental healthcare needs, especially among vulnerable groups such as children and adolescents.

**Aim:**

This study examined changes in the number of overall and diagnosis-specific mental health ED visits among patients aged <18 years following the onset of the COVID-19 pandemic in Madrid, Spain.

**Methods:**

Using clinical records from all psychiatric ED visits at a major teaching hospital between October 2018 and April 2021, we conducted interrupted time-series analyses and compared trends before and after the day of the first ED COVID-19 case (1st March 2020).

**Results:**

A total of 663 patients were included. In March 2020, there was a marked initial decrease of -12.8 (95% CI -21.9, - 7.9) less monthly mental health ED visits. After April 2020, there was a subsequent increasing trend of 3.4 (95% CI 2.6, 4.2) additional monthly mental health ED visits.

**Conclusion:**

After the onset of the COVID-19 pandemic, there was an increase in paediatric psychiatric ED visits, especially due to suicide-related reasons. These data reinforce the crucial role of the ED in the management of acute mental health problems among youth and highlight the need for renovated efforts to enhance access to care outside of and during acute crises during the pandemic and its aftermath.

## Introduction

The COVID-19 pandemic is having a substantial negative mental health impact on the population across the globe (1). A large body of evidence indicates that children and adolescents are especially vulnerable to the negative mental health effects of major population stressors, such as epidemics, armed conflicts, or natural disasters (2, 3, 4). In addition, certain stressors brought about by the initial phases of the COVID-19 pandemic, such as disruption of in-person schooling (5), may have been particularly distressing for children and adolescents and could relate to the reported increased feelings of loneliness, despair, depressive and anxiety symptoms (6,7).

Emergency Departments are an important pathway to care for children and adolescents with mental health problems (8), especially in the presence of barriers in access to outpatient resources. During the initial COVID-19 outbreak, health system reorganization efforts to cope with abruptly increased needs in acute medical care led to limited outpatient healthcare delivery (Amador et al. 2021) – especially in major initial COVID-19 hotspots like Madrid (9), certain areas of Italy (10) or the United States (11). Notwithstanding, initial evidence from these areas suggested that the number of psychiatric emergency department visits dropped drastically during the initial phases of the pandemic – generating concern that the increase in mental health needs was not being met (12). Notably, the availability of timely accessible mental health care is paramount to reduce the burden of psychiatric disorders and mental health-related disability (13).

Recent evidence indicates sustained negative mental health outcomes among children and adolescents during the first year of the COVID-19 pandemic (14). However, data on psychiatric emergency department visits beyond the initial pandemic outbreak remain largely unexplored – despite implications as a potential indicator of mental healthcare provision adequacy (10). The main aim of this study was to analyze changes in the number of child and adolescent psychiatric emergencies between the pre- COVID-19 period and the year following the outbreak in Madrid, a major COVID-19 hotspot, characterizing the frequency and trends in specific reasons for child and adolescent psychiatric emergency department consultation.

## Methods

### Participants and procedure

We used electronic health records to extract the monthly numbers of total and diagnosis-specific mental health ED visits among patients aged <18 years, between October 2018 and April 2021, to La Paz University Hospital, a large teaching hospital providing acute and emergency care to a catchment area of around 525,000 people in Madrid, Spain, one of the largest global pandemic hotspots during Spring 2020.

Diagnoses were exacted from ED clinical records and were grouped into the following DSM-5 diagnostic categories: Depressive disorders, anxiety disorders, schizophrenia spectrum and other psychotic disorders and eating disorders. Given the prevalence of suicide-related phenomena as a main complaint in ED, we added them as a specific category, including suicidal ideation or suicide attempt (defined as any self-injurious act with at least some intent to die based on the psychiatrist clinical judgment).

Of the total of 663 patients included, 67.5% were females. The mean age was 15 years and two months. The most prevalent diagnostic category was suicide-related phenomena, which accounted for 43.2% of ED visits, followed by depressive disorders (21.0%), anxiety disorders (19.2%), schizophrenia spectrum and other psychotic disorders (6.6%), and eating disorders (2.5%).

### Analyses

We conducted interrupted time-series analyses (15) to quantify trends in the monthly number of patients seen in the Psychiatric Emergency Department and to compare trends before and after the initial COVID-19 pandemic outbreak (March 2020). For this purpose we employed the itsa command in Stata v.16 for PC (16), which is based on OLS regression models designed to adjust for autocorrelation typically observed in time series (17).

### Results

Interrupted time-series analyses indicated that mental health ED visits among patients aged <18 years remained overall stable between October 2018 and February 2020 (β 0.2; 95% CI -0.2, 0.6). In March 2020, there was a marked initial decrease of -12.8 (95% CI -21.9, -7.9) monthly mental health ED visits. After April 2020, there was a subsequent increasing trend of 3.4 (95% CI 2.6, 4.2) additional monthly mental health ED visits. The difference in trends before and after March 2020 was of 3.2 (95% CI 2.3, 4.1) additional monthly mental health ED visits.

Diagnostic-specific analyses suggested that overall increases in mental health ED visits were driven by increases in suicide-related phenomena (β: 2.0; 95% CI 1.1, 2.8 visits per month). Following the initial pandemic outbreak, we also detected increases in anxiety (β: 0.73; 95% CI -0.03, 1.10) and depressive disorders (β: 0.77; 95% CI -0.34, 1.20) – though for the latter the wide confidence intervals are also compatible with decreasing trends. There were hardly any differences in eating disorders (β 0.07; 95% CI -0.05, 0.18) and schizophrenia spectrum and other psychotic disorders (β: 0.04; 95% CI -0.07, 0.15). (See Additional file 1: Table 1 and Figure A.1 for detail)

**Figure 1:**
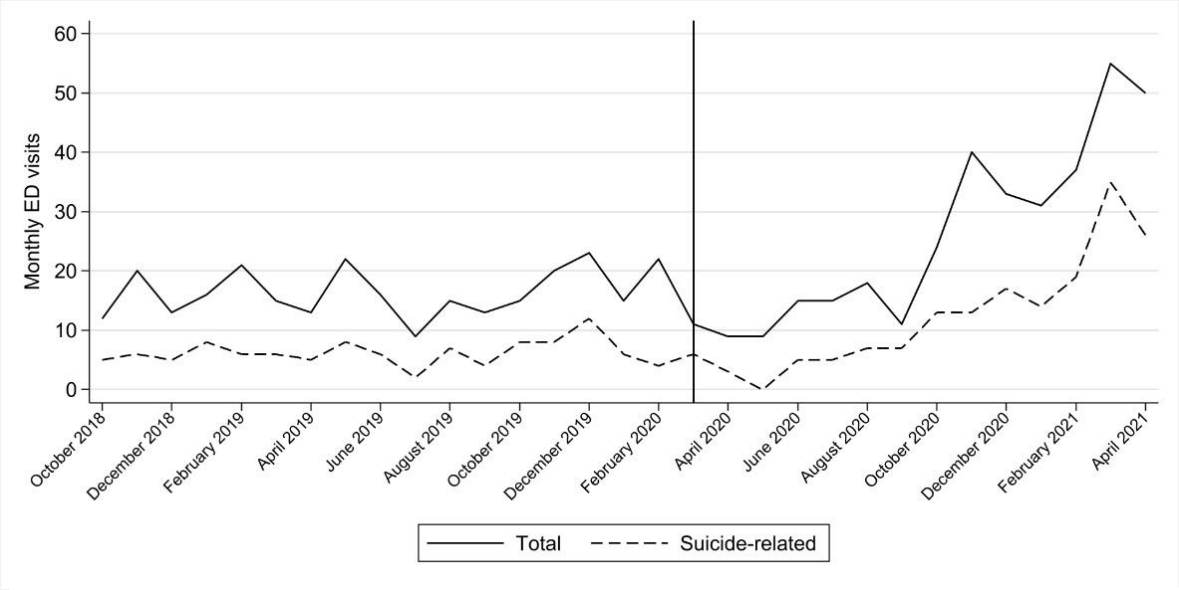
Monthly children and adolescents Psychiatric Emergency Department Visits. Vertical line indicates March 2020, the beginning of the COVID-19 pandemic in Madrid.

### Discussion

Mental health ED visits among children and adolescents decreased sharply shortly after the initial COVID-19 outbreak, and subsequently increased over the following months - with over three more monthly mental health ED visits between April 2020 and April 2021. Other studies reported a similar trend, with a decrease in mental health visits to ED during lockdown compared to the year before, followed by an increase in the reopening months (18).

Furthermore, during the post-lockdown period, there was an increase in diagnoses of suicidal behavior (19) and, to a lesser extent, of affective (20) and anxiety disorders (18). Several factors may explain this phenomenon. First, while mental health ED visits decreased during the initial pandemic outbreak – mainly due to fear of contagion and generalized lockdown measures – population-based studies indicate that mental health distress and suicidal ideation, were already on the rise (21). It seems plausible that mental health ED visits only increased once the fear of contagion and lockdown measures progressively faded away. Second, children and adolescents may be particularly vulnerable to generalized stressors related to the pandemic – such as death of loved ones, family distress due to emerging economic problems, and loss of peer support networks due to lockdown measures and online schooling (22,23). Other authors have reported that the number of patients diagnosed with schizophrenia and eating disorders visiting the emergency department remained relatively unchanged after COVID-19 (19,20). Whether this is due to no increases in exacerbations of these disorders after the pandemic outbreak, exacerbations not presenting to the ED, or lack of power to detect changes in trends due to a small sample size, remains unanswered.

We identified a sustained increase in child and adolescent mental health ED requirements during the months following the initial pandemic onset. These findings highlight the importance of strengthening child and adolescent mental healthcare provision in the context of the ongoing pandemic and its aftermath. Particular emphasis should be placed in ensuring access to interventions to prevent suicide and suicidal behaviors, as the most salient increase in child and adolescent mental health ED visits corresponded to suicide-related consultations. Similar increases in youth ED visits or hospital admissions due to suicide attempts during the initial months of the pandemic period were seen in Turkey (24), the United States (25), and elsewhere in Spain (26).

Most evidence-based suicide prevention interventions for youth, such as the Safety Planning Intervention or dialectical-behavioral therapy (27,28), can be performed using telepsychiatry resources or on the telephone. Accordingly, expanding telematic child and adolescent mental healthcare delivery seems like a reasonable response to increases in mental healthcare needs as it would, at the same time, enhance access to suicide prevention strategies and lower the burden on hospitals’ EDs.

Our study has limitations that should be considered. As with most studies based on electronic healthcare records, our study is limited by potential errors in the transcription of clinical information. However, we used broad, easy-to-detect diagnostic categories, and there is no reason to believe that presence of such errors may have varied over the study period. Also, by focusing solely on ED visits, we are underestimating the actual increase in mental health needs brought about by the pandemic, as we could only detect the most severe cases (29), including heavy suicide attempters with a tendency to recidivism and repeated ED visits (30).

Even though the study center’s catchment area is a large, highly heterogeneous area with over half a million inhabitants, our results may not be representative of the entire population of the region of Madrid. Our data, however, remain valid to estimate the increase in pediatric psychiatric emergency needs, and we hope that it promotes further analysis of this issue in larger populations.

In conclusion, shortly after the onset of the COVID-19 pandemic, there was an increase in pediatric psychiatric ED visits, especially due to suicide-related reasons. These data reinforce the crucial role of the ED in the management of acute mental health problems among youth and highlight the need for renovated efforts to enhance access to care outside of and during acute crises during the pandemic and its aftermath.
